# Links between Psychotropic Substance Use and Sensation Seeking in a Prevalence Study: The Role of Some Features of Parenting Style in a Large Sample of Adolescents

**DOI:** 10.1155/2014/962178

**Published:** 2014-09-21

**Authors:** Marco Scalese, Olivia Curzio, Valentina Cutrupi, Luca Bastiani, Mercedes Gori, Francesca Denoth, Sabrina Molinaro

**Affiliations:** Institute of Clinical Physiology-Italian National Research Council (IFC-CNR), 56124 Pisa, Italy

## Abstract

*Aims*. The objectives of the study were to (a) investigate the prevalence risk of current drug users and (b) explore the association between parental monitoring, adolescent-parent relationship, family structure, financial status, and sensation-seeking and psychotropic substance use. *Methods*. Data were drawn from the 2002 Italian student population survey of the European School Survey Project on Alcohol and Other Drugs. The sample size was 10,790 adolescents, aged 15–19 years. Multivariate logistic analyses were performed. * Findings*. The prevalence of users was 27.3% (34.2% males; 21.6% females). Single-parent and reconstructed families were related to the greatest likelihood of substance use. A medium financial status and, for females, a satisfying relationship with father were protective factors. Probability of engaging in risk-taking behavior increased when parental knowledge decreased. Exploring deeper how parental monitoring could modify the relation between different traits of sensation seeking and substances use revealed the following: “thrill and adventure seeking,” within the case of a good monitoring, can help against the use of substances; “boredom susceptibility” is not associated with drug use, except when parental monitoring is weak. *Conclusions*. Specific subdimensions, associated with substance use, may be more amenable to prevention than general interventions on sensation-seeking personality. Family is the context that could promote health education.

## 1. Introduction

Drug use is a widespread and expanding epidemic among high school students [1–4]. Substance-related behavior in adolescence is influenced by temperamental, psychological, and social factors. Social environmental factors include also parenting styles and the related parental risk behaviors modeling [5–7]. Personality characteristics such as SS are strongly associated with drug, tobacco, and alcohol use and misuse in adolescents [8, 9] so that the assessment of it could be particularly useful in regard to prevention of substances abuse in teenagers. Sensation seeking, a personality trait with biological roots, is associated with the need for novel, complex, and intense sensations and experiences and the willingness to take physical, social, legal, and financial risks for the sake of such experiences [10].

Regarding potential risk factors, it has been suggested that family structure could mediate the expression of temperamental risk of substance use behavior [11]. During the last decade, family structure has changed. Between 2000 and 2010, in Italy, for every 1000 marriages the divorce rate increased from 114.9 to 181.7 [12]. The percentage of intact families decreased from 41.2% to 37.2%, the single-parent family rate rose from 7.7% to 8.1%, and the “reconstituted families” rate (formed after the end of a previous conjugal union of at least one of the partners) increased as well (from 4.7% to 6.1%) [13]. Family adversity has been associated with an increased risk of substance use disorders [14] and family structure could influence parenting styles (parental monitoring and the adolescent-parent relationship) and also buffer the expression of the temperamental trait related to substance use behavior [5, 6, 15].

The quality of adolescent-parent relationship is an important topic in adolescent addiction research because the relational environment that parents create and develop through parental socialization (i.e., parenting) has been hypothesized to be one of the strongest predictors of youth and young adult substance abuse (e.g., [16]). A significant number of studies have investigated the predictors of initiation, trajectories, and severity of youth and young adults' substances abuse. Predictors examined have included also parental influences (e.g., [17, 18]).

Little consensus exists regarding the relationship between families' socioeconomic status (SES) and substance use. For example, the study by Patrick et al. [18] examined the associations of family socioeconomic status during childhood with smoking, alcohol use, and marijuana use during young adulthood. Smoking in young adulthood was associated with lower childhood family socioeconomic status, although the association was explained by demographic and social role covariates. Alcohol use and marijuana use in young adulthood were associated with higher childhood family SES, even after controlling for covariates. Families' socioeconomic status is linked with family structure because single parent family, for example, seems to have more socioeconomic problems (economic resource explanation of family structure effects). Family structure is in turn related to parental monitoring; single-parent families would provide insufficient resources for adequate adolescent socialization and control. A variety of studies outlined that part of the family structure effect is attributable to parent-child relation and differences in family income and this influenced parental monitoring too (e. g. [11, 19]).

Parents play an important role in terms of the development of their adolescent offspring [20, 21] and a number of researchers in the field of behavioural genetics have been reporting on the combinations and interactions between parental behaviours, heritability, and shared environmental influences (e.g., [22, 23]). The influence of parents has been highlighted by several studies [21–34]. Some of these researches highlighted in particular the importance of positive fathering—suggesting the unique importance of fathers in relation to externalizing and internalizing behaviours [6]. Despite the increased distance between children and parents during adolescence, the quality of the parent-adolescent relationship matters, even for adolescents beginning the transition to adulthood [20].

Such as in other studies the objective of this one is to explore how each factor reported above and financial family status lead to substance use behavior. Few studies explore the interactions of SS with parental knowledge: research convincingly shows that adolescents are influenced considerably by specific parenting practices like monitoring and control [25].

The beneficial effects of parental monitoring on substance use/abuse/dependence could be varied also on the base of age [26]; high parental monitoring in particular during first years of high school can decrease the extent of an adolescent's affiliation with substance-using peers and decreasing affiliation with substance-using peers might instill in adolescents the importance of having appropriate nonusing friends, beliefs, and skills. Monitoring of children's and adolescence's behavior is considered an essential parenting accomplishment. Well-monitored youths are less involved in norm breaking and risk behaviors. Parents could get knowledge from their children's spontaneous disclosure of information and their own active surveillance efforts. It is important in this context also taking into account that authoritarian and authoritative parenting styles can differently affect adolescent outcomes (see [27–30]). More precisely are the parental warmth dimension and parental socialization that would represent the major features associated with lower levels of substance use [28, 30–34].

The present themes have implications for prevention. Parents might be able to reduce the risk for substance use by preventing initiation during high school through monitoring and involvement. Continuing parental monitoring and having conversations on a wide range of topics and also about the dangers of alcohol and drug use might be needed. Parent-based interventions, which aim to enhance parental communication and support, parent-child relationship, and specific parent-teen dialog, regarding the risks of substances use and abuse, have shown initial success in reducing the risk of drug abuse. Understanding how parental monitoring acts warrant more investigation. Identifying the relation between parental monitoring and sensation seeking to buffer the risk for dependence and identifying sensation seeking as a way to target adolescents at risk for substance use are a focus in prevention research [35–37]. Parents should have an active role in identifying risk factors in their children that are associated with increased risk for substance use disorders [26].

In a large sample of adolescents, this study investigated the prevalence of current drug users (any use during the previous month of psychoactive substances) in adolescents and explored the association between parental monitoring, adolescent parent relationship, family structure, financial status, and sensation-seeking and substance use behavior. Given the strong evidence that adolescents with high sensation-seeking tendencies use more psychotropic substances, the literature lacks on why some sensation seekers do not engage in psychotropic substance use. We studied this phenomenon analyzing the association between sensation-seeking, drug use, and moderation role of parental control and how it can modulate the relation between different traits of SS with substances use. If we can determine that some features of parenting style as protective factors offset the negative role of sensation seeking as a risk factor, then there may exist many opportunities to address parents with specific information about their parenting style that will help mitigate or protect against their children's risk for drug use.

## 2. Method

### 2.1. Recruitment

Sampling and data collection procedures are summarized here; full details are available in the 2007 European School Survey Project on Alcohol and Other Drugs (ESPAD) Report [38]. Data collection was performed by standardized methodology using self-administered questionnaires completed in the classroom, and participation was completely voluntary. In Italy there is no need to perform any scientific ethical review in order to collect ESPAD data. Sampled schools were contacted to enable the teacher responsible for health education to present the research project to the school board. The parents had to be informed via passive consent. The students were informed that their participation would be on an anonymous basis. All relevant national ethical rules were followed in the performance of the study. The target population comprised Italian high-school students aged 15–19 years [1]. The questionnaire targeted alcohol, tobacco, and cannabis use and included simple questions on the sociodemographic situation of the adolescent. After the compilation of the standard ESPAD-Italia 2002 questionnaire, a subsample of students was asked to fill out an additional module composed of Sensation Seeking Scale (SSS) [39]. The subsample of 13,000 subjects was randomly chosen from the original sample of ESPAD-Italia 2002 questionnaire and there were no significant differences between this subsample and the original larger sample. Of 13,000 ESPAD subsample students, 10,790 fully responded to the additional modules (45.3% males; mean age = 17.1, SD = 1.5) and there were no significant differences between individuals who fully responded to the additional questionnaires and those who did not.

#### 2.1.1. Dependent Variable: Drug Use

We defined as current users those adolescents who had used psychoactive substances during last month (LM) (tranquillizers or sedatives without a medical prescription and cannabis, cocaine, heroin, stimulants, and hallucinogens) and students who experienced heavy episodic drinking (5 or more drinks on a single occasion) ≥3 times in LM and smoked ≥11 cigarettes per day during LM; LM frequency was included to assess persistence of a general pattern [40].

#### 2.1.2. Independent Variables

Personal variables included age, gender, SSS scores, and family features. The SSS was designed to assess the personality traits of thrill and adventure seeking, disinhibition, experience seeking, and susceptibility to boredom. For the present study the 1971 revised version of Zuckerman et al.'s original measure [41] was used. As reviewed by Roberti [42] the SSS is a valid and reliable method for determining an individual's behavioral expression of sensation-seeking traits. The SSS consists of 40 items, each having two options from which the participant must choose one. The SSS provides a score for four subscales and one total score. The subscales are as follows: (1) thrill and adventure seeking (TAS): desire to engage in sports or activities involving speed and danger; (2) disinhibition (Dis): desire for social and sexual disinhibition; (3) experience seeking (ES): desire for experience through the mind and senses, travel, and nonconformist lifestyle; (4) boredom susceptibility (BS): aversion to repetition and routine. Scores of each subscale were taken into account to better explore how total score is influenced by them. In fact, total score could be affected by individual subscale score, generating a misrepresentation of the relationship between SSS and health risk behavior [43].

Family characteristics: (1) parental monitoring or knowledge was addressed in the question “My parents know where I am on Saturday night” (4-point scales from “Always know” to “Never know”). As in the paper of Kokkevi et al; (2) adolescent-parent relationship was assessed for mother and father by 5-point scales from “very satisfied” to “dissatisfied”; (3) adolescent family structure as intact, reconstituted or single-parent family was addressed in the question “With whom do you live?”; (4) financial status was evaluated by the answer in a 7-point scale to the question “What is the economic status of your family compared to others?”, from “very much above” to “very much below” [44].

### 2.2. Statistical Analyses

#### 2.2.1. Descriptive Analyses

Categorical variables are expressed as percentages. Comparisons between groups were made using a chi-square test. The reliability of the SSS (total and subscales scores) was assessed by Cronbach's alpha.

#### 2.2.2. Multivariate Analysis

Multivariate logistic regression analysis was performed in order to assess the association between psychoactive substances and sensation-seeking, age, sex, parental monitoring, adolescent-parent relationship, family structure, and financial status.

All models (numbered from 1 to 7) include demographic variables, family variables, and total score of SSS or four subscales. The final models were stratified by age and gender (Models 4 and 5) and by family monitoring (Models 6 and 7). Results are reported as adjusted odds ratio (OR) and 95% confidence interval. Data were analyzed using SPSS version 17.

## 3. Results

### 3.1. Descriptive Analyses

#### 3.1.1. Students' Drug Use

The prevalence of psychotropic substance users among adolescents was 27.3% (34.2% males; 21.6% females). Drug use prevalence increased with age: peak age was 19 years (37.5% overall; 46.8% males and 29.6% females). Cannabis, alcohol, and tobacco are the substances most commonly used by young people; in particular heavy drinking episodes (13.3% LM) and joint consumption of alcohol and cannabis (12.3% LM) were observed. Tranquilizers (without medical prescription), cocaine, and heroin (especially smoked, 1.0%) were used by a lower percentage of students (Table 1).

#### 3.1.2. Students' Social Background

Characteristics of the entire sample and of drug and nondrug users are reported in Table 2 for boys and girls. The majority of the sample lived in intact families (89.0%). A significant percentage of adolescent users were dissatisfied with their relationship with parents (*P* < 0.001), and perceived parental monitoring or knowledge was significantly lower in drug consumers (*P* < 0.001). Age, economic family status, and family structure were related to drug use as well (*P* < 0.001) (Table 2).

#### 3.1.3. Sensation Seeking Traits in Substance Users and Nonusers Adolescents

Global Sensation seeking mean scores and subscales mean scores were all significantly different for substance users adolescents (*P* < 0.001). The SSS total and subscale scores, for all samples and for the subpopulations stratified by gender, are summarized in Table 3. Drug users had higher SS scores than nonusers: they displayed a higher total score and higher subscale score (Table 3).

### 3.2. Internal Consistence of the SSS

The internal consistencies of the subscales of the SSS as measured by Cronbach's alpha were for the TA 0.73, for the ES 0.45, for the Dis 0.67, and for the BS subscale 0.44. Cronbach's alpha for the total SSS score was 0.80.

### 3.3. Binary Logistic Models to Assess the Association between Drug Use and SS Traits and Social-Relational Features

#### 3.3.1. Univariate Analysis (Table 4, Model 1)

Parental monitoring showed a kind of gradient in the probability of using psychotropic drugs in comparison with students who state that their parents always know where they spend Saturday evening; the probability of using drugs was about three, four, and five times higher for those who declared that their parents know, respectively, often, seldom, and never where they spend the evening (never OR = 5.229, 95% CI [4.444–6.152]).

#### 3.3.2. Multivariate Analysis with SSS Total Score (Table 4, Model 2)

Students living in single-parent and reconstructed families showed a higher probability of using drugs than others (OR = 1.628, 95% CI [1.341–1.977] and OR = 1.512, 95% CI [1.076–2.125] resp.). Also in this case, the lower the level of parental monitoring observed, the more the adolescents were prone to drug use. A satisfying relationship with each parent was an important issue in this context.

#### 3.3.3. Multivariate Analysis with SSS Subscales (Table 4, Model 3)

The experience seeking (OR = 1.349, 95% CI [1.304–1.395]) and the disinhibition (OR = 1.361, 95% CI [1.326–1.398]) subscales showed interesting values in the probability of engaging in drug consumption: for each subscale point the probability of observing this behavior increased by 30%.

### 3.4. Stratified Multivariate Analysis to Assess Drug Use in Young and Old Adolescents of Both Gender

#### 3.4.1. Multivariate Analysis by Gender and Age (Models 4 and 5)

Exploring differences in drug use according to demographic characteristics, data were stratified for age and gender to identify variables that influence the most drug use in each subgroup, first by observing the risk using the total score of SSS (Table 5 and Model 4), secondly by exploring the role of subscales (Table 5 and Model 5).

An overview of results shows variables which transversely characterized all subsamples. For all subsamples, age (only for the young adolescents) and parental monitoring seemed to be the most influential variables in drug consumption. The likelihood of engaging in consumption of substances was similar among males and females aged 15–17 years (around 45% for each additional year). Sensation-seeking trait was also related to substance consumption. More precisely, disinhibition (Dis) and experience seeking (ES) were the subscales involved in the likelihood of engaging in drug consumption behavior. On the contrary, thrill and adventure seeking (TAS) subscale was not at all related to drug consumption. Family structure was not a significant variable associated with drug use in males but it was especially important for younger females. Moreover, for female subgroups, the father's positive emotional support was a protective factor.

For young males (15–17 years), the variable that increased the likelihood of drug use the most was parental knowledge. The probability of engaging in drug behavior was up to three times greater when parental monitoring was lower (*often* parents know: OR = 1.592, 95% CI [1.316–1.927];* seldom* parents know: OR = 2.435, 95% CI [1.915–3.097];* never* parents know: OR = 3.229, 95% CI [2.379–4.38]). Moreover, for each year the probability increased by about 47% (OR = 1.468, 95% CI [1.359–1.584]). In the matter of sensation seeking the likelihood of drug use increased to 23% and 39% for each point on the Dis and ES subscales, respectively.

Also for older males (18-19 years) the likelihood of drug use rose when monitoring by parents occurred* seldom* (OR = 1.649, 95% CI [1.158–2.349]). The strength of the association between drug use and monitoring was weaker compared to the younger males. Regarding sensation-seeking, the likelihood of taking drugs increased about 18% for each point of the total score. In particular, also in this case adolescents characterized by higher scores in the Dis and ES subscale were more prone to problematic behavior (for DIS: OR = 1.399, 95% CI [1.284–1.524]; for ES: OR = 1.344, 95% CI [1.256–1.439]).

For young females (15–17 years), age increased the likelihood of drug use by about 44% each year. Both living in a reconstructed family and in a single-parent family increased the probability of drug use up to twofold (OR = 1.781, 95% CI [1.066–2.976]; OR = 2.092, 95% CI [1.550–2.822]). The relation to parental knowledge was strong (*often* parents know: OR = 1.649, 95% CI [1.344–2.023];* seldom* parents know: OR = 2.507, 95% CI [1.920–3.274];* never* parents know: OR = 1.843, 95% CI [1.284–2.645]). In regard to sensation seeking, in this subsample as well the likelihood of drug use rose by 19% for each point of the scale. In this subsample ES and Dis increased the probability of drug use by 42% and 34%, respectively, as did the BS scale by about 6% for each point of the scale.

Results for older female adolescents (18-19 years) were similar. In this group as well, the strongest risk factor seemed to be parental monitoring. The likelihood of taking drugs rocketed to twofold when monitoring decreased, compared to* always* controlled adolescents (*often*: OR = 1.759, 95% CI [1.303–2.375];* seldom*: OR = 1.671, 95% CI [1.119–2.495];* never*: OR = 2.23, 95% CI [1.319–3.77]). Living in single-parent families was also strongly related to drug behavior (OR = 1.759, 95% CI [1.045–2.962]). The SSS had the same strength of association with drug use as the other subsamples (OR = 1.196, 95% CI [1.163–1.230]) also for Dis and ES subscales (for DIS: OR = 1.552, 95% CI [1.405–1.714]; for ES: OR = 1.357, 95% CI [1.268–1.452]).

### 3.5. Stratified Multivariate Analysis to Assess Drug Use for Different Levels of Family Monitoring

#### 3.5.1. Stratified Multivariate Analysis for Family Monitoring (Models 6 and 7)

**Figure 1:**
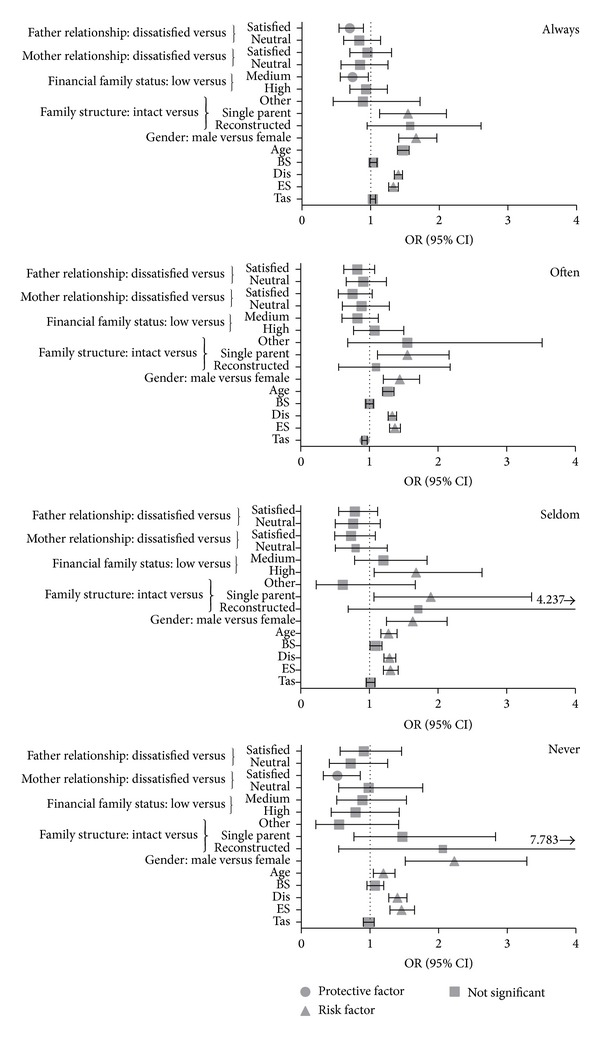
Multivariate logistic model for substance use stratified for parental control with SSS subscales (Model 7).

Data stratified according to family monitoring permitted exploration of how drug use was related to the variables of interest, in particular sensation seeking, adolescent-parent relationship, and family structure. SSS (for Dis and ES traits) and age were transversely significant in risk of engaging in substance use regardless of monitoring by the family (see Table 6 and Figure 1). In particular, the likelihood of drug use was about 20% for each point of the SSS independently of having strict monitoring or not. Regarding gender, in males the risk of substance use was always higher; particularly, it was twice that for females when monitoring never occurred (OR = 2.144, 95% CI [1.487–3.092]) and 70% more when monitoring was* always* (OR = 1.707, 95% CI [1.455–2.004]). With reference to age, the likelihood of engaging in substance or alcohol use was always significant, especially when monitoring was* always* (OR = 1.490, 95% CI [1.408–1.575]). In single-parent families, the risk of engaging in a behavior of substance use increased up to twofold when monitoring was lower (*seldom*: OR = 1.991, 95% CI [1.126–3.518]). When monitoring occurred* seldom* in families where the economic status was higher, the probability of substance use was 70% greater with respect to a lower socioeconomic status. On the other hand, protective factors appeared to be strict monitoring occurring in families where the relationship with the father was satisfying and in middle-class families. Another protective factor appeared to be the case when monitoring* never* occurred but satisfying emotional support from the mother was reported (see Figure 1).

## 4. Discussion

Given the strong evidence that adolescents with high sensation-seeking tendencies use more psychotropic substances, the literature lacks on why some sensation seekers do not engage in substance use. Although parental monitoring seems to have a definite advantage in protecting against substance use, it was not strong enough to prevent or buffer sensation seeking effect as risk factor for substance use; in fact, the relation between this personality trait and excessive substance use appeared invariable regarding the level of parental monitoring. For this reason we chose to explore deeper how parental monitoring could modify the relation between different traits of SSS and substances use. The possibility to better investigate about different subdimensions of the trait revealed interesting differences: specifically “thrill and adventure seeking” within the case of a good monitoring on behalf of parents can be a good help against the use of substances. Moreover, boredom susceptibility, which seems to be generally associated with drug use and above all among young girls, when stratifying on the base of parental monitoring it is interesting to find out that, is not generally associated except in the case when parental monitoring is not so strong. Potentially those specific subdimensions, identified in our study as associated with substance use, may be more amenable to prevention than a general prevention on sensation-seeking personality: such data would be very informative and are recommended for future research.

We examined a population of 10,790 high-school students and data confirmed the well-known association between sensation-seeking behavior and drug use [8, 45], but this factor did not seem to represent the unique feature related to risk behavior if compared to the other considered variables. In particular we found that parental supervision had an important role in the behavioral expression of sensation-seeking traits, in this case excessive drug use and alcohol and tobacco misuse. As with many other factors or variables, sensation seeking is a continuum and a matter of degree. The higher the level of sensation seeking, the greater the likelihood of drug abuse; the lower the level of parental monitoring, the greater the risk of psychotropic substances consumption.

According to the biopsychosocial perspective, demographic, temperamental, and social variables influence substance use behavior. Furthermore, although all these factors are individually important, it is likely that they are interrelated and act together, rather than separately, to influence health behavior [46].

Sensation seeking (SS) is an easily measured personality trait, providing valuable information about preferences for risky and nonrisky forms of arousal. Although SS did not represent the heaviest variable within the models, it remained stable and significant in all the analyses. These findings could confirm that sensation seeking and its aspects belong to the category of personality characteristics with few differences between males and females as stated in literature [47, 48]. The internal consistencies of the subscales as measured by Cronbach's alpha were also similar to those found in the original [49] and in Fortune and Goodie [43] studies especially for the thrill and adventure seeking (TAS) subscale, for the disinhibition (Dis) subscales, and for the total SSS score. The internal consistencies for the other subscales were lower than those seen in the previous studies reported above.

Results could contribute to the debate regarding the possibility of two separate constructs as suggested in literature. The first, composed by disinhibition (Dis) and boredom susceptibility (BS) scales, focuses on past or current behavior and feelings regarding the SS experiences. The second, made up of thrill and adventure seeking (TAS) and experience seeking (ES) scales, focuses on the desire to engage in SS activities [43]. The subscales that mostly characterized the subsamples of drug users were ES and Dis. These results confirm previous data in adolescence literature where Dis, ES, and SSS total score are the measures most associated with all drug categories' use [2].

In our study for all adolescents the stronger the perceived parental control monitoring, the lower the substances use. This relation seemed to be stronger in females and younger males, who were more “family-oriented” than older males. This observation could be related to some cultural characteristics of the Mediterranean countries in which girls, even after 17 years, are more controlled and kept close to the family than boys of the same age. Males, even if they are monitored by the family, feel more independent than girls.

As previously stated in literature the most effective prevention programs are “family-centered” [50]. Moreover family environment, specifically family support, parenting practices, and parent/adolescent relationships are empirically established predictors of adolescent drug problems [7] and also of adolescent drug treatment success [51]. Older male adolescents seemed to be less conditioned by the family. On the other hand, contrary to the findings of Choquet and colleagues [5], parental knowledge levels showed a kind of gradient in the probability of use of psychotropic drugs.

Expanding the logic argued by Patock-Peckham and colleagues [6], parental monitoring is crucial in early adolescence; in our findings for the younger compared to the older adolescents, the likelihood of drug use increased progressively when monitoring was less. Parental control monitoring and quality of relationship with parents were used as an indicator of a more general family support and communication. Adolescent-parent relationship was a very important variable in all the models performed. Regarding the role of the father, in particular for younger females but also for the oldest ones, the results of our research confirmed the opposite-gender parent's effect on behavioral monitoring [6, 52]. Families can be characterized by two different aspects: the normative and the affective. The first, usually related to the father's role, would promote the adjustment to social rules; the second, more connected to the mother, fosters emotional development. The interactions between the two codes will determine the family's educational style, which will influence adolescents' personality factors. Keeping parental control monitoring attitudes and adolescent-parent relationships as a concise measure of the “state of health” of the family, our data may support the positive effect of the paternal role in relation to adaptive behavior. There have been several evidences to suggest that the emotional interactions between mothers and their children differ from those of fathers. Thus, it appears that mothers and fathers contribute to the development of “different modes of affective sharing and coregulation” [53]. In a Flemisch study De Groof and Smits [54] found that the association between family type and problem behaviour disappeared after controlling for parental monitoring by the father. A good father-child relationship and high parental monitoring predict a lower level of delinquency among male and female adolescents. The relationship with the mother seems to be less important in explaining the differences in delinquency among adolescents. Moreover in single-parent families a good relationship with the biological father is associated with less drunkenness among adolescents [21, 24]. At the same time mothers who endorse nonsupportive emotion socialization strategies have children that are emotionally dysregulated and inexpressive in the social context [55].

In single-parent and reconstructed families, offspring were more attracted to the consumption of psychoactive substances, as previously reported by Miller [19] and Hoffmann [11]. Furthermore, to our data this is true for younger females and, in the specific case of a single-parent family, also for the older ones, is possibly due to a poor father-daughter relationship. Usually, in Italy the offspring of separated/divorced unions live with the mother. An interesting datum is that stratifying for parental monitoring only single-parent families shows an increased risk of developing problem drug use. This propensity for drug use could be explained in different ways. For instance, often low-income single-parent families provide poorly for an adolescent's adequate socialization and monitoring [11]. On the other hand, families with a higher socioeconomic status provide adolescents with greater financial means, which could lead younger ones to come into contact with drugs unless monitored. Those considerations are supported by our data, where a middle-class family seemed to be a protective context.

Several limitations must be kept in mind considering this study. First of all the study design is not a prospective one and does not evaluate causal relationships between any risk conditions and drug use; on the other hand it offered the possibility for generalization due to the wide and representative sample analyzed. Another limitation is that this survey involved only adolescents attending high school thus it could probably underestimate the prevalence of substance use among the Italian young population.

Although the standard questions of the ESPAD survey allow evaluating the frequency and pattern of psychoactive substance use and the quantity of alcohol in centiliters, it does not allow calculating the quantity in grams neither for each substance nor for alcohol. Meanwhile, self-reports [56] are the most eligible tools for obtaining accurate information about excessive substance consumption among adolescents due to the anonymity and confidentiality. They have the strength to avoid observer bias; nevertheless they have the disadvantage of the possible presence of self-reporter bias.

Another limitation of this study is that the quality of parental relationship with the adolescent and parental monitoring are only concise measures of parenting style, of the real situation in the family, and of the relationship of each parent with the child; even more there is to say that, although this method of measuring parental monitoring has been used in prior research [57], it would have been more helpful if we got data from parents as well. Unfortunately we did not have dyadic data and so relied on adolescents report on parental relationship and monitoring even knowing that adolescents and parents give discrepant reports about parents' rearing behaviors [58]. Parenting or educational styles should be evaluated by standardized scales as questionnaires like the EMBU which allow a double independent evaluation for children and parents [59].

The SSS-V is a questionnaire that contains 4 items on direct consumption of alcohol and other drugs and therefore it would be suitable for further investigation of the use of recently developed questionnaires (without drugs items) to measure sensation seeking trait, as, for example, the ZKAPQ [60]. Meanwhile the present ESPAD survey could be useful in the context of a concordance and consistency study. Furthermore, there are many other issues that have not been analyzed in this research, for instance, the availability of legal and illegal substances, the peer-group characteristics, and the circumstances where the adolescent is located.

Despite the above mentioned limitations, this study has made some important contributions to the literature. First of all, since psychotropic substance use is a highly significant problem among adolescents, it seems appropriate to well understand what factors are associated with their use. Much more, although research suggests that both parental monitoring and levels of sensation seeking are associated with psychotropic substance use, most of them looked at their direct effect and few examined their interactive effects. This is extremely important given that most studies look at the independent effects of these predictors without giving thought to other potential relationships. Future development in this area can present ideas on the best way to monitor children with different trait of sensation seeking and impulsivity. Kaynak et al. [26] have conducted similar research. Kaynak and colleagues found that parental monitoring did not moderate the relationship between sensation seeking and substance use on the development of substance use. The current investigation differs from Kaynak study and adds to the literature an indication of the moderating role of parental monitoring on the influence of sensation seeking trait. In fact the value of the association between substance use and sensation seeking in the multivariate model number 2 (OR = 1.80, 95% CI [1.167–1.193]) is different from the value of the association when the model is stratified by parental monitoring and it is less strong only than the value of the association only respect to the low level of parental monitoring (parents always know OR = 1.202, 95% CI [1.181–1.223]; parents often know OR = 1.150, 95% CI [1.129–1.171]; parents seldom know OR = 1.174, 95% CI [1.142–1.207]; parents never know OR = 1.199, 95% CI [1.155–1.245]).

Given the fact that SSS results may help identify adolescents prone to consumption of substances, monitoring that is linked to emotional support could be modified in order to decrease the risk and increase the protective parental function, respectively. These results can be achieved by operating on communication and affective styles of communication. Evidence regarding age, gender, and family feature differences in the epidemiology of drug use may be an important consideration for a family-oriented community prevention strategy, considering teenage peer culture as well [61, 62].

Early identification of risky behavior and attitudes in adolescents and replacing them with nonrisky alternative ones is essential for reducing negative health and social consequences. It is desirable to improve most novelty seekers' potential abilities to pursue their divergent thinking in the production of art and science, through participation in action-adventure [63] or more generally providing peer resistance techniques and social alternative activities [64].

Epidemiological surveillance plays a key role in the detection of specific risky features of the phenomenon of drugs misuse that can be modified. To achieve an efficient health promotion program, in addition to a policy of health, it is fundamental to identify individuals, groups, and communities able to acquire knowledge and learn skills indispensable for making decisions about their health. In this case, families are the context that could promote health education and thus changes in social relationships and social rules as well.

## Figures and Tables

**Table 1:** Prevalence of drug, alcohol, and tobacco use among adolescents according to age, psychotropic substance, and gender.

	Male %	Female %	All %
Age			
15 years	16.4	10.7	13.3
16 years	25.8	16.8	21.0
17 years	35.8	22.8	28.8
18 years	42.3	25.0	32.3
19 years	46.8	29.6	37.5
Last 30 days' substance use			
Tobacco (≥10 cigarettes)	7.7	5.3	6.4
Heavy drinking	19.5	8.1	13.3
Cannabis	20.4	13.2	16.5
Solvents	2.8	0.9	1.8
Tranquilizers	0.9	2.4	1.7
Amphetamine	0.7	0.3	0.5
LSD	0.8	0.3	0.5
Crack	0.5	0.1	0.3
Cocaine	1.9	1.0	1.4
Heroin smoked	1.4	0.7	1.0
Heroin	0.3	0.0	0.2
Ecstasy	1.0	0.4	0.7
Steroids	0.6	0.1	0.3
Any other drugs	0.3	0.1	0.2
Alcohol + pills	1.3	0.5	0.8
Alcohol + cannabis	15.7	9.5	12.3
Total	**34.2**	**21.6**	**27.3**

**Table 2:** Sample features and description of substance users and substance nonusers characterized by the main sociodemographic variables according to gender.

Characteristics	All sample			Drug users			Nondrug users			*P* value		
	Male	Female	All	Male	Female	All	Male	Female	All	Male	Female	All
	%	%	%	%	%	%	%	%	%			
Age										<0.001	<0.001	<0.001
15 years	18.3	18.5	18.4	8.8	9.2	9.0	23.3	21.1	22.0			
16 years	19.6	18.1	18.8	14.8	14.0	14.5	22.1	19.2	20.4			
17 years	19.2	18.8	19.0	20.1	19.8	20.0	18.7	18.5	18.6			
18 years	17.7	20.0	19.0	21.9	23.1	22.4	15.6	19.1	17.7			
19 years	25.2	24.6	24.9	34.4	33.8	34.1	20.3	22.1	21.4			
Financial family status										0.005	<0.001	<0.001
High	36.2	27.0	31.2	30.2	36.2	32.8	55.1	65.8	61.4			
Medium	55.8	62.7	59.6	33.5	34.9	34.1	28.5	22.8	25.1			
Low	8.0	10.4	9.3	22.1	19.0	20.8	11.2	7.6	9.1			
Parental control										<0.001	<0.001	<0.001
Always	46.5	59.4	53.6	14.2	9.9	12.3	5.3	3.8	4.4			
Sometimes	30.2	25.4	27.6	38.1	29.1	34.2	35.2	26.4	30.0			
Seldom	15.0	10.1	12.3	52.8	58.1	55.1	57.4	64.0	61.3			
Never	8.3	5.1	6.6	9.1	12.8	10.7	7.4	9.7	8.7			
Mother relationship										<0.001	<0.001	<0.001
Dissatisfied	7.0	12.2	9.8	11.0	20.3	15.0	4.9	10.0	7.9			
Neutral	10.2	13.6	12.1	13.3	16.0	14.4	8.7	12.9	11.2			
Satisfied	82.8	74.2	78.1	75.7	63.8	70.5	86.5	77.1	80.9			
Father relationship										<0.001	<0.001	<0.001
Dissatisfied	10.7	18.9	15.2	15.0	30.0	21.5	8.4	15.9	12.8			
Neutral	12.1	16.6	14.5	16.3	17.3	16.7	9.9	16.4	13.7			
Satisfied	77.3	64.5	70.3	68.7	52.7	61.8	81.7	67.7	73.4			
Family structure										0.006	<0.001	<0.001
Intact	89.9	88.2	89.0	87.9	81.4	85.1	91.0	90.0	90.4			
Reconstructed	1.5	2.2	1.9	1.6	3.5	2.5	1.4	1.8	1.6			
Single parent	6.9	8.4	7.7	8.8	13.2	10.7	5.9	7.1	6.6			
Other	1.7	1.3	1.4	1.7	1.8	1.8	1.8	1.1	1.3			

All samples	4,885	5,905	10,790	1,671	1,275	2,946	3,214	4,630	7,844			

**Table 3:** Sensation Seeking Scale (SSS) global and subscale mean scores (M) and standard deviation (SD) for the entire sample, for substance users and nonusers adolescents, and for gender.

	All sample						Substance users						Nonsubstance users					
	Male		Female		All		Male		Female		All		Male		Female		All	
	M	SD	M	SD	M	SD	M	SD	M	SD	M	SD	M	SD	M	SD	M	SD
SSS	20.3	5.0	18.5	5.4	19.3	5.3	22.8	4.7	22.4	5.0	22.6	4.8	19.0	4.7	17.4	4.9	18.1	4.9
TAS^a^	6.1	2.1	5.8	2.2	5.9	2.2	6.3	2.0	6.3	2.1	6.3	2.1	5.9	2.2	5.6	2.3	5.7	2.2
ES^a^	5.4	1.7	5.5	1.7	5.5	1.7	6.1	1.7	6.7	1.6	6.4	1.7	5.0	1.6	5.2	1.5	5.2	1.6
Dis^a^	4.5	2.2	3.3	2.3	3.8	2.3	5.6	2.0	4.9	2.3	5.3	2.1	3.9	2.0	2.9	2.0	3.3	2.1
BS^a^	4.4	1.7	3.8	1.6	4.1	1.7	4.8	1.7	4.5	1.7	4.7	1.7	4.2	1.6	3.7	1.6	3.9	1.6

^a^Subscale scores: TAS: thrill and adventure seeking; Dis: disinhibition; ES: experience seeking; BS: boredom susceptibility.

All tests are significant with *P* < 0.001.

**Table 4:** Binary logistic models for substance use (Models 1, 2, and 3).

Variables in the equation	Univariate^a^ OR (CI 95%)	Multivariate SSS total score^b^ OR (CI 95%)	Multivariate SSS subscales^c^ OR (CI 95%)
Sensation Seeking Scale total score	1.209 (1.197–1.221)∗	1.180 (1.167–1.193)∗	—
TAS^d^	1.134 (1.112–1.157)∗	—	0.992 (0.968–1.016)
ES^d^	1.586 (1.541–1.633)∗	—	1.349 (1.304–1.395)∗
Dis^d^	1.522 (1.489–1.556)∗	—	1.361 (1.326–1.398)∗
BS^d^	1.332 (1.298–1.367)∗	—	1.041 (1.007–1.075)∗
Age	1.364 (1.323–1.407)∗	1.372 (1.323–1.422)∗	1.350 (1.301–1.401)∗
Gender: male versus female	1.888 (1.733–2.057)∗	1.597 (1.442–1.768)∗	1.602 (1.440–1.782)∗
Family structure: intact family versus			
Reconstructed	1.623 (1.215–2.168)∗	1.512 (1.076–2.125)∗	1.471 (1.035–2.092)∗
Single parent	1.732 (1.495–2.006)∗	1.628 (1.341–1.977)∗	1.590 (1.304–1.939)∗
Other	1.415 (0.951–2.105)	0.967 (0.648–1.444)	0.868 (0.575–1.312)
Parental control: always versus			
Often	2.537 (2.290–2.811)∗	1.532 (1.364–1.720)∗	1.481 (1.315–1.667)∗
Seldom	4.230 (3.721–4.807)∗	2.181 (1.882–2.527)∗	1.910 (1.642–2.221)∗
Never	5.229 (4.444–6.152)∗	2.233 (1.846–2.701)∗	2.032 (1.672–2.470)∗
Financial family status: low versus			
High	0.937 (0.804–1.092)	1.039 (0.866–1.247)	1.059 (0.879–1.276)
Medium	0.735 (0.635–0.850)∗	0.832 (0.700–0.989)∗	0.840 (0.704–1.001)
Mother relationship: dissatisfied versus			
Neutral	0.750 (0.626–0.898)∗	0.861 (0.699–1.060)	0.858 (0.693–1.062)
Satisfied	0.586 (0.505–0.680)∗	0.798 (0.671–0.949)∗	0.783 (0.656–0.935)∗
Father relationship: dissatisfied versus			
Neutral	0.778 (0.666–0.910)∗	0.823 (0.687–0.986)∗	0.841 (0.699–1.012)
Satisfied	0.613 (0.540–0.696)∗	0.761 (0.654–0.885)∗	0.774 (0.664–0.903)∗

^a^Model 1. ^b^Model 2. ^c^Model 3. ^d^Subscales score: TAS: thrill and adventure seeking; Dis: disinhibition; ES: experience seeking; BS: boredom susceptibility.

∗ *P* < 0.05.

**Table 5:** Multivariate logistic model for substance use for gender and age.

Variables in the equation	Females 15–17 years	Females 18-19 years	Males 15–17 years	Males 18-19 years
	OR (95% CI)	OR (95% CI)	OR (95% CI)	OR (95% CI)
Model 4				
Sensation Seeking Scale^a^	1.198 (1.175–1.222)∗	1.196 (1.163–1.230)∗	1.152 (1.131–1.174)∗	1.184 (1.150–1.218)∗
Age	1.447 (1.335–1.568)∗	1.229 (0.674–2.240)	1.468 (1.359–1.584)∗	0.682 (0.209–2.230)
Family structure: intact versus				
Reconstructed	1.781 (1.066–2.976)∗	1.282 (0.541–3.038)	1.456 (0.789–2.685)	1.230 (0.369–4.101)
Single parent	2.092 (1.550–2.822)∗	1.759 (1.045–2.962)∗	1.373 (0.965–1.953)	1.068 (0.638–1.787)
Other	0.703 (0.252–1.964)	1.947 (0.844–4.489)	0.592 (0.298–1.178)	1.360 (0.552–3.354)
Parental control: always versus				
Often	1.649 (1.344–2.023)∗	1.759 (1.303–2.375)∗	1.592 (1.316–1.927)∗	0.983 (0.726–1.331)
Seldom	2.507 (1.920–3.274)∗	1.671 (1.119–2.495)∗	2.435 (1.915–3.097)∗	1.649 (1.158–2.349)∗
Never	1.843 (1.284–2.645)∗	2.230 (1.319–3.770)∗	3.229 (2.379–4.383)∗	1.322 (0.847–2.063)
Financial family status: low versus				
High	1.084 (0.794–1.480)	0.870 (0.546–1.387)	1.204 (0.861–1.685)	0.721 (0.465–1.119)
Medium	0.880 (0.660–1.174)	0.765 (0.503–1.165)	0.826 (0.594–1.149)	0.778 (0.517–1.170)
Mother relationship: dissatisfied versus				
Neutral	0.759 (0.554–1.039)	0.902 (0.537–1.517)	1.050 (0.696–1.584)	0.736 (0.404–1.341)
Satisfied	0.750 (0.578–0.973)	0.860 (0.560–1.321)	0.851 (0.604–1.199)	0.807 (0.485–1.344)
Father relationship: dissatisfied versus				
Neutral	0.737 (0.559–0.972)∗	0.613 (0.391–0.961)∗	1.053 (0.731–1.519)	1.030 (0.634–1.675)
Satisfied	0.747 (0.591–0.943)∗	0.773 (0.538–1.110)	0.791 (0.583–1.072)	0.861 (0.582–1.274)

Variables in the equation	Females 15–17 years	Females 18-19 years	Males 15–17 years	Males 18-19 years
	OR (95% CI)	OR (95% CI)	OR (95% CI)	OR (95% CI)
Model 5				

TAS^b^	1.016 (0.973–1.061)	0.999 (0.937–1.066)	0.965 (0.926–1.005)	0.976 (0.917–1.040)
ES^b^	1.421 (1.338–1.509)∗	1.552 (1.405–1.714)∗	1.237 (1.172–1.305)∗	1.399 (1.284–1.524)∗
Dis^b^	1.327 (1.267–1.390)∗	1.357 (1.268–1.452)∗	1.398 (1.336–1.463)∗	1.344 (1.256–1.439)∗
BS^b^	1.062 (1.002–1.125)∗	0.966 (0.881–1.060)	1.028 (0.976–1.084)	1.086 (0.998–1.181)
Age	1.412 (1.300–1.533)∗	1.289 (0.704–2.359)	1.448 (1.338–1.566)∗	0.711 (0.219–2.301)
Family structure: intact versus				
Reconstructed	1.765 (1.031–3.022)∗	1.164 (0.474–2.855)	1.506 (0.806–2.814)	0.962 (0.288–3.208)
Single parent	1.934 (1.423–2.630)∗	1.864 (1.088–3.193)∗	1.375 (0.957–1.974)	1.129 (0.670–1.902)
Other	0.572 (0.194–1.683)	1.816 (0.741–4.449)	0.502 (0.250–1.005)	1.422 (0.564–3.585)
Parental control: always versus				
Often	1.525 (1.238–1.879)∗	1.814 (1.329–2.476)∗	1.591 (1.308–1.934)∗	0.894 (0.653–1.223)
Seldom	2.235 (1.702–2.934)∗	1.400 (0.922–2.127)	2.052 (1.602–2.628)∗	1.493 (1.035–2.153)∗
Never	1.696 (1.169–2.461)∗	2.038 (1.183–3.510)∗	3.026 (2.212–4.141)∗	1.124 (0.715–1.769)
Financial family Status: low versus				
High	1.141 (0.830–1.569)	0.819 (0.506–1.327)	1.214 (0.863–1.706)	0.728 (0.464–1.142)
Medium	0.909 (0.677–1.220)	0.720 (0.467–1.112)	0.836 (0.598–1.168)	0.765 (0.504–1.162)
Mother relationship: dissatisfied versus				
Neutral	0.745 (0.540–1.029)	0.873 (0.508–1.501)	1.089 (0.716–1.655)	0.789 (0.429–1.452)
Satisfied	0.707 (0.542–0.924)∗	0.819 (0.525–1.276)	0.883 (0.623–1.253)	0.873 (0.518–1.469)
Father relationship: dissatisfied versus				
Neutral	0.768 (0.579–1.018)	0.620 (0.389–0.987)∗	1.064 (0.733–1.544)	1.016 (0.619–1.667)
Satisfied	0.774 (0.610–0.982)∗	0.780 (0.535–1.138)	0.768 (0.563–1.047)	0.890 (0.594–1.333)

^a^Total score. ^b^Subscales score: TAS: Thrill and Adventure Seeking; Dis: Disinhibition; ES: Experience Seeking; BS: Boredom Susceptibility.

∗ *P* < 0.05.

**Table 6:** Multivariate logistic model for substance use for parental control.

Variables in the equation	Always	Often	Seldom	Never
	OR (95% CI)	OR (95% CI)	OR (95% CI)	OR (95% CI)
Model 6				
Sensation Seeking Scale^a^	1.202 (1.181–1.223)∗	1.150 (1.129–1.171)∗	1.174 (1.142–1.207)∗	1.199 (1.155–1.245)∗
Age	1.490 (1.408–1.575)∗	1.296 (1.217–1.381)∗	1.297 (1.187–1.417)∗	1.245 (1.098–1.412)∗
Gender: male versus female	1.707 (1.455–2.004)∗	1.390 (1.169–1.653)∗	1.612 (1.247–2.084)∗	2.144 (1.487–3.092)∗
Family structure: intact versus				
Reconstructed	1.554 (0.945–2.557)	1.128 (0.590–2.156)	1.772 (0.727–4.322)	3.001 (0.841–10.704)
Single parent	1.547 (1.142–2.094)∗	1.618 (1.174–2.228)∗	1.991 (1.126–3.518)∗	1.480 (0.780–2.809)
Other	0.912 (0.475–1.751)	1.664 (0.756–3.661)	0.636 (0.236–1.710)	0.768 (0.306–1.924)
Financial family status: low versus				
High	0.941 (0.708–1.251)	0.992 (0.718–1.371)	1.708 (1.095–2.664)∗	0.753 (0.424–1.337)
Medium	0.730 (0.557–0.957)∗	0.801 (0.590–1.088)	1.254 (0.826–1.906)	0.838 (0.496–1.418)
Mother relationship: dissatisfied versus				
Neutral	0.783 (0.532–1.153)	0.894 (0.622–1.287)	0.827 (0.528–1.295)	1.060 (0.596–1.886)
Satisfied	0.911 (0.672–1.236)	0.786 (0.575–1.073)	0.789 (0.537–1.160)	0.527 (0.329–0.846)∗
Father relationship: dissatisfied versus				
Neutral	0.820 (0.601–1.119)	0.880 (0.649–1.194)	0.747 (0.496–1.125)	0.691 (0.398–1.198)
Satisfied	0.687 (0.535–0.881)∗	0.794 (0.612–1.031)	0.768 (0.541–1.088)	0.924 (0.583–1.465)

Variables in the equation	Always	Often	Seldom	Never
	OR (95% CI)	OR (95% CI)	OR (95% CI)	OR (95% CI)
Model 7				

TAS^b^	1.033 (0.995–1.073)	0.929 (0.890–0.970)∗	1.016 (0.955–1.082)	0.980 (0.903–1.063)
ES^b^	1.333 (1.264–1.406)∗	1.372 (1.295–1.453)∗	1.308 (1.206–1.418)∗	1.461 (1.293–1.651)∗
Dis^b^	1.405 (1.348–1.465)∗	1.334 (1.275–1.396)∗	1.295 (1.212–1.385)∗	1.401 (1.274–1.540)∗
BS^b^	1.040 (0.988–1.095)	1.002 (0.947–1.060)	1.092 (1.009–1.183)∗	1.072 (0.957–1.200)
Age	1.475 (1.393–1.562)∗	1.273 (1.193–1.359)∗	1.280 (1.168–1.403)∗	1.196 (1.049–1.365)∗
Gender: male versus female	1.664 (1.409–1.965)∗	1.444 (1.203–1.733)∗	1.633 (1.250–2.133)∗	2.231 (1.515–3.285)∗
Family structure: intact versus				
Reconstructed	1.576 (0.951–2.611)	1.097 (0.553–2.177)	1.712 (0.692–4.237)	2.061 (0.546–7.783)
Single parent	1.545 (1.133–2.105)∗	1.554 (1.118–2.161)∗	1.894 (1.067–3.364)∗	1.473 (0.767–2.829)
Other	0.887 (0.457–1.720)	1.552 (0.685–3.517)	0.613 (0.225–1.670)	0.549 (0.212–1.420)
Financial family status: low versus				
High	0.934 (0.699–1.246)	1.076 (0.771–1.502)	1.681 (1.069–2.641)∗	0.790 (0.437–1.428)
Medium	0.736 (0.559–0.968)∗	0.825 (0.602–1.130)	1.203 (0.786–1.841)	0.890 (0.517–1.532)
Mother relationship: dissatisfied versus				
Neutral	0.845 (0.569–1.255)	0.885 (0.607–1.292)	0.798 (0.505–1.262)	0.982 (0.545–1.770)
Satisfied	0.955 (0.698–1.306)	0.755 (0.547–1.042)	0.733 (0.495–1.087)	0.525 (0.321–0.859)∗
Father relationship: dissatisfied versus				
Neutral	0.834 (0.608–1.145)	0.910 (0.663–1.248)	0.764 (0.504–1.158)	0.719 (0.411–1.260)
Satisfied	0.696 (0.540–0.897)∗	0.823 (0.628–1.077)	0.788 (0.553–1.122)	0.909 (0.565–1.462)

^a^Total score. ^b^Subscales score: TAS: thrill and adventure seeking; Dis: disinhibition; ES: experience seeking; BS: boredom susceptibility.

∗ *P* < 0.05.
